# Cardiac Autonomic Neuropathy in Newly Diagnosed Patients With Type 2 Diabetes Mellitus

**DOI:** 10.7759/cureus.47366

**Published:** 2023-10-20

**Authors:** Md Sabah Siddiqui, Vishnu Dev, Ekta Khandelwal

**Affiliations:** 1 General Medicine, All India Institute of Medical Sciences, Raipur, Raipur, IND; 2 Physiology/Autonomic & Vascular Functions, All India Institute of Medical Sciences, Raipur, Raipur, IND

**Keywords:** autonomic function, chhattisgarh, india, diabetes mellitus, cardiac autonomic neuropathy

## Abstract

Introduction: Cardiac autonomic neuropathy (CAN) is a debilitating complication in diabetes mellitus, leading to life-threatening arrhythmias and various impairments. Its prevalence varies widely, and early detection and management are crucial. This study investigates the prevalence of CAN in newly diagnosed type 2 diabetes mellitus patients in Central India, comparing them to a control group.

Methodology: This case-control study included 35 newly diagnosed type 2 diabetes mellitus patients and 35 age-matched healthy controls from the general population. Cardiac autonomic function testing (AFT) was done by heart rate variability (HRV), the deep breathing test (DBT), the cold pressor test (CPT), and the lying-to-standing test (LST). Parameters were recorded and analyzed using statistical tests.

Results: Patients with type 2 diabetes mellitus had significantly higher weight, BMI, fasting blood sugar, post-prandial blood sugar, urine albumin-creatinine ratios, and systolic and diastolic blood pressure than controls. Abnormalities in HRV and E: I ratio during the DBT and CPT were more prevalent in these patients. Parasympathetic dysfunction (delta HR) and a lower E: I ratio were also significantly high in this group. Also, LST results suggested a greater likelihood of orthostatic symptoms in the patients' group.

Conclusion: This study highlights the importance of AFT in diagnosing early CAN in newly diagnosed patients. Early diagnosis and management of diabetic CAN are essential to prevent complications.

## Introduction

Cardiac autonomic neuropathy (CAN) is one of the debilitating complications in patients with diabetes mellitus. It comprises damage to the autonomic nerve fibres, which innervate the heart. It predisposes patients to life-threatening arrhythmias, silent myocardial infarction, and other features impairing daily activities, like orthostatic hypotension, resting tachycardia, exercise intolerance, and intraoperative hemodynamic compromise [1]. This entity is often overlooked in diabetic patients as the features of subtle autonomic impairment might not be clinically evident until it hampers the patient’s life quality. With the increase in the duration of diabetes, there is an increased risk of complications associated with the illness, both macro and micro-vascular.

The prevalence of CAN in diabetes mellitus patients has been reported to be between 7.7% and 90% [2,3] depending upon the study methodology and the characteristics of the study population. It is well known that early disease detection and strict glycaemic control, pharmacotherapy, and lifestyle modifications can reduce the morbidity of this disease.

A significant risk factor in developing CAN is the duration of diabetes in both type 1 and type 2 diabetes mellitus [4]. In similar studies conducted in newly diagnosed diabetes mellitus patients, the prevalence of cardiac autonomic neuropathy was around 28-32% [5,6].

The association between CAN and major cardiac events in diabetes mellitus patients has been studied, showing a significant association between both [7]. Indians, having a different phenotype of diabetes compared to the Caucasian population, are much more prone to the illness's early-onset insulin resistance and complications. However, very few studies have been conducted in India on CAN, and some studies have shown an earlier onset of CAN in the Indian population [8], which can be correlated with the so-called Asian-Indian phenotype of diabetes [9].

This study aims to determine the prevalence of CAN in newly diagnosed diabetes mellitus patients attending a tertiary care centre in Central India and compare the findings to a control group.

## Materials and methods

The present article is a case-control study conducted on patients admitted to general medicine OPD, AIIMS Raipur, and referred to the autonomic vascular function laboratory, AIIMS Raipur. The study participants include patients (18-45 years of age) with type 2 diabetes mellitus within the last year. Patients who denied approval or had a history of coronary artery disease, renal disease or cerebrovascular disease were excluded from the study.

The sample size was calculated using the formula (n= Z^2^pq/e^2^). In the present study, the total Cases and controls included were 35 in each group. Those diagnosed with Type 2 diabetes mellitus based on the ADA Diagnostic Criteria of Type 2 Diabetes Mellitus, aged 18-45, who attend the General Medicine OPD of AIIMS Raipur, were taken as cases. At the same time, healthy age-matched subjects from the general population with no comorbidities were consented to the study were included as controls.

After getting the proper consent, brief personal, medical and drug histories were taken. Patients were informed about the procedure of autonomic function testing (AFT). Patients underwent heart rate variability (HRV) testing, the lying-to-standing test (LST), the cold pressor test (CPT), and the deep breathing test (DBT) at AFT Laboratory. The values were interpreted for autonomic dysfunction (Table 1). For all protocols, ECG, pulse and respiration were recorded on an eight-channel digital physiograph and software (Lab chart, ADI, Australia).

**Table 1:** Details for the procedure of AFT testing

Tests	Protocol	Recording
Heart Rate Variability (HRV)	Recording of HRV was monitored using a lead II electrocardiogram (ECG) in the supine position for five minutes. HRV analysis was done using the same software with dedicated modules.	
Autonomic Function Tests		
Deep Breathing Test (DBT)	The patient was instructed to breathe smooth, deep, and slow. Controlled inspiration for 5 sec and expiration for 5 seconds for six cycles per minute was done.	Continuous recording of ECG and respiration was taken for six cycles. The calculation for heart rate changes was done from the tracing of respiration and ECG.
Cold Pressor Test (CPT)	Baseline BP was taken, and then the patient was instructed to keep the hand up to the wrist immersed in cold water at 10 degree Celsius water for 1 minute and immediately indicate to the investigator if they are uncomfortable. After the hand is removed from the water, it is covered by a towel.	Baseline blood pressure and ECG were recorded. Blood pressure was again taken just before the hand was taken out of the water (i.e., in the end, 1 minute of immersion). Blood pressure was retaken at 1.5 min and 4 min after the hand was withdrawn from the cold water.
Lying-to-Standing Test (LST)	The patient was instructed about the test. The test was conducted after 10 min of supine rest. Then, he was told to assume the standing posture within the count of 3 seconds, and recordings were taken.	Blood pressure and heart rate were recorded at baseline and serially at 0.5th, 1st, 2nd, 2.5th and 5th min. A 30:15 ratio was calculated from ECG.

After obtaining the parameters, offline analysis was done. Statistical parameters were analysed using IBM SPSS Statistics for Windows, Version 26 (Released 2019; IBM Corp., Armonk, New York, United States). For statistical significance, each parameter was tested for data distribution using the Kolmogorov-Smirnov test of normality. If the data distribution was “Gaussian”, parametric tests like independent t-test or Welch’s paired ‘t-test were applied. An appropriate non-parametric test like the Mann-Whitney test was used for non-Gaussian distribution. The statistical difference was considered significant at a two-tailed p-value of<0.05. Data is represented as mean with standard deviation or median with quartiles.

## Results

In this study, 35 patients and 35 controls were enrolled. Cases and controls were matched by gender, age, and height. Their demographic profile was compared, and it was found that patients had a significantly higher mean weight and BMI than controls. Physiological parameters showed that the patients had higher fasting blood sugar, post-prandial blood sugar, and urine albumin-creatinine ratios than controls. Systolic blood pressure was significantly higher in patients, as was diastolic blood pressure. However, heart rates did not differ significantly between groups (Table 2).

**Table 2:** Demographic profile and baseline physiological parameters Values shown are mean ±S.D., p-value* ≤ 0.05 (significant) ACR: Albumin-creatinine ratio

Parameter	Patients (n=35) [Mean+/- Standard Deviation]	Controls (n=35) [Mean+/- Standard Deviation]	p-value
Age (years)	40.08 ± 4.42	40 ± 4.65	0.9414
Height (cm)	158.39 ± 7.19	158.17 ± 5.83	0.8886
Weight (kg)	65.03 ± 12.39	59.84 ± 7.95	0.0401*
BMI (kg/m^2^)	26.03 ± 4.89	23.88 ± 3.10	0.0318*
Hba1c	8.03 ± 1.88	5.32 ± 0.40	<0.00001*
Fasting blood sugar	124.82 ± 25.48	79.77 ± 7.04	<0.00001*
Postprandial blood sugar	175 ± 54.91	104.74 ± 8.07	<0.00001*
Urine ACR	42.20 ± 44.4	9.05 ± 2.74	<0.00001*
Systolic blood pressure (mmHg)	127.37 ± 12.76	121.42 ± 7.78	0.014*
Diastolic blood pressure (mmHg)	82.65 ± 7.39	74.62 ± 6.30	0.0001*
Heart rate (beats per minute)	80.91 ± 13.59	76.14 ± 8.50	0.0828

Regarding cardiovascular reflex tests, patients showed significantly lowered HRV, expiration RR Interval: insipration RR Interval ratio (E: I ratio) during the DBT, CPT, and LST as compared to controls. Parasympathetic dysfunction, indicated by delta HR and E: I ratio, was significantly more prevalent in patients than controls (Table 3).

**Table 3:** Frequency of normal and abnormal cardiovascular reflex tests among the patients and controls Values shown in percentage %

Parameter	Patients (n=35)			Controls (n=35)		
	Number of subjects	Normal (%)	Abnormal (%)	Number of subjects	Normal (%)	Abnormal (%)
Heart rate variability (P)	35 (100%)	4 (11.4%)	31 (88.66%)	35 (100%)	29 (83%)	6 (17%)
E: I ratio during deep breathing (P)	35 (100%)	24 (68.6%)	11 (31.4%)	35 (100%)	30 (85.7%)	5 (14.3%)
Cold pressor test (S)	32 (91%)	8 (22.9%)	24 (68.6%)	35 (100%)	31 (88.6%)	4 (11.4%)
Lying-to-standing test (S)	35 (100%)	30 (85.7%)	5 (14.3%)	35 (100%)	32 (91.4%)	3 (8.6%)

All the parameters of AFT were significantly decreased in patients compared to controls. Parasympathetic markers like root mean square of successive differences (RMSSD) and standard deviation of the RR interval (SDRR) of HRV were significantly lower in patients. The total power of HRV was lower in patients. The LF/HF ratio was lower in patients but did not reach statistical significance (Table 4).

**Table 4:** Comparison of AFT and HRV parameters between patients and controls Values are shown in Median (1 quartile – 3 quartile), p-value* ≤ 0.05 (significant). AFT: Autonomic function; HRV: heart rate variability; RMSDD: root mean square of successive differences; SDRR: standard deviation of the RR interval

Parameter	Patients (n=35)	Controls (n=35)	p-value
ΔHR (bpm)	21.11 (14.859-28.92)	30.2 (26.04-38.7)	0.00038*
E: I Ratio	1.311 (1.193-1.430)	1.425 (1.274-1.662)	0.00714*
LST ΔSBP (mmHg)	1 (-4 – 10)	12 (10 – 14)	<0.0001*
CPT ΔSBP (mmHg)	1.5 (-4 – 5.75)	12 (10 – 15)	<0.0001*
SDRR (ms)	27.4 (16.25-50.12)	40.23 (34.37-49.51)	0.02574*
RMSSD (ms)	22.1 (9.61-36.15)	35.89 (20.325-41.25)	0.01352*
LF/HF	1.22 (0.64-2.59)	1.73 (1.29-2.13)	N.S.
Total Power	292.47 (155.5-1116.50)	1289.98 (599.24-2385.37)	0.036*

## Discussion

Indian studies on CAN in newly diagnosed type 2 diabetes mellitus patients are limited. One survey by Jyotsna et al. found abnormal cardiac autonomic function in 71% of diagnosed diabetic patients, compared to 33.76% of controls [10]. Their mean HbA1c was 7.7 ± 1.7 gm/dl, while our study had a mean HbA1c of 8.03 ± 1.88 gm/dl, possibly indicating delayed diagnosis and poor control.

Bhuyan et al. reported a CAN prevalence of 70% in diabetic individuals with an average diabetes duration of 9.03 ± 6.4 years and a mean HbA1c of 10.9 ± 15.5 gm/dl. The most common abnormal test was the E: I Ratio, found in 56% of their patients, compared to 31% in our study [11]. Khandelwal et al. found that 80.64% of people with diabetes had CAN using Ewing's criteria, dropping to 42.7% with Bellavere's criteria [3]. In a study by Aggarwal et al., the prevalence of CAN in diabetic patients attending a tertiary care centre in Central India was 70%. The study population consisted of patients with a means duration of diabetes of 5.89±4.57yrs. Also, they included both type 1 diabetes mellitus and type 2 diabetes mellitus patients [12].

Nijhawan et al. studied 25 type 2 diabetes mellitus patients for CAN and found that 60% of the study population had CAN and diabetic cystopathy in 68% of the individuals [13]. Ratzmann et al. studied the prevalence of neuropathy and cardiac autonomic functions in 95 newly diagnosed diabetes patients. He used Valsalva manoeuvre, LST and DBT to assess the autonomic functions. The study revealed a prevalence of 7.3% of CAN in newly diagnosed diabetics. This low prevalence could have been due to the absence of HRV and other standardised tests to identify CAN at that point [14].

In a study conducted by Mohan et al. on a cohort of 336 patients with type 2 diabetes mellitus, autonomic dysfunction was 35.7%. In this study, the mean duration of diabetes was 9.3 ± 6.1 years, and the mean HbA1c was 9.7 ± 2.1 gm/dl. A lower prevalence of autonomic dysfunction in this study can be attributed to the selection of stringent diagnostic criteria whereby at least two or more heart rate tests must be abnormal to tell that a patient has definite CAN [15].

Ziegler et al. have found that the prevalence of CAN in newly diagnosed diabetic individuals ranges from 17% to 22%, depending upon the number of abnormal tests considered together. Their study consisted of a cohort containing type 1 diabetes mellitus and type 2 diabetes mellitus patients aged 11-72 years, with an HbA1c of 8.9 gm/dl [16].

The higher CAN prevalence in our study might be due to the faster progression of diabetes in the Indian population, which was reflected in higher baseline HRV abnormality. The Asian-Indian diabetes phenotype is associated with increased insulin resistance and a greater risk of micro and macrovascular complications, contributing to these differences. So, early diagnosis of diabetic-associated autonomic neuropathy can prevent the development of comorbidities and can provide better longevity without complications. 

## Conclusions

This study assessed the prevalence of CAN in early-diagnosed type 2 diabetes mellitus patients in Central India. Our findings indicate a relatively high prevalence of CAN in this population, with a significant proportion of patients exhibiting abnormalities in various autonomic function tests, including HRV, the DBT, and the cold pressor test. This highlights the importance of early detection and monitoring of CAN in diabetic patients, especially in regions with a high prevalence of diabetes, like India. These results emphasise the need for proactive management strategies to prevent the development of complications associated with CAN in diabetic patients, which can significantly impact their quality of life and overall health.
